# Dynamics of Gut Microbiota in Japanese Tits (*Parus minor*) Across Developmental Stages: Composition, Diversity, and Associations with Body Condition

**DOI:** 10.3390/microorganisms13122840

**Published:** 2025-12-14

**Authors:** Li Zhang, Lele Kang, Keping Sun, Longru Jin, Haitao Wang

**Affiliations:** 1 Jilin Engineering Laboratory for Avian Ecology and Conservation Genetics, Northeast Normal University, Changchun 130024, China; zhangl082@nenu.edu.cn (L.Z.); kangll457@nenu.edu.cn (L.K.); wanght402@nenu.edu.cn (H.W.); 2 Jilin Provincial Key Laboratory of Animal Resource Conservation and Utilization, Northeast Normal University, Changchun 130117, China; sunkp129@nenu.edu.cn

**Keywords:** gut microbiota, Japanese tit, temporal shifts, SMI

## Abstract

The gut microbiota forms early in life and undergoes dynamic changes that are essential for host health and development. Although body condition is a key fitness-related trait and predictor of viability in many animal species, its association with gut microbiota, especially during early life stages in wild populations, remains poorly understood. In this study, we collected fecal samples and used 16S rRNA gene sequencing to investigate temporal shifts in gut microbiota of Japanese tits (*Parus minor*) across nestling stages (days 3, 6, 10, and 14 post-hatching, denoted as D3, D6, D10, and D14, respectively; n = 70, repeatedly sampled) and in adults (n = 25), and examined their association with nestling body condition. The results showed that nestlings harbored distinct microbial communities compared to adults, with the latter exhibiting higher relative abundances of Bacteroidota and Verrucomicrobiota (LMMs, *p* < 0.001 for both). LEfSe analysis identified Actinobacteriota as a biomarker for D14 nestlings and Bacteroidota for adults. Alpha diversity decreased from D3 to D14, with adults showing higher diversity than late-stage nestlings (D10–D14), but comparable diversity to early-stage nestlings (D3–D6). Beta diversity revealed clear separation between nestlings and adults, and within nestlings, early stages (D3–D6) differed significantly from D14. Nest effects also contributed to microbial structure. Time-lagged analyses showed that Firmicutes abundance at D6 positively predicted scaled mass index (SMI) at D10 (*p* = 0.002), whereas Proteobacteria negatively predicted SMI (*p* = 0.006). Overall, these findings highlight dynamic, stage-specific shifts in the gut microbiota of Japanese tits and suggest that microbial succession may influence nestling growth and physiological adaptation.

## 1. Introduction

The gut microbiota, a complex and dynamic community of microorganisms residing in the host digestive tract, plays a crucial role in host health, fitness, and survival [1,2,3]. It contributes to a wide range of physiological functions, including nutrient absorption [4], trace metal detoxification [5], immune regulation and pathogen defense [6,7,8], and even influences brain physiology and behavior [9]. Beyond individual-level benefits, gut microbiota can influence broader ecological and evolutionary traits, such as energy allocation, reproductive success, and longevity [10,11,12]. Multiple factors shape these communities, including diet [13,14], genetics [15,16], social interactions [17,18], and environmental conditions [19,20], with host age emerging as a particularly influential factor [21,22]. Early-life colonization patterns can have lasting effects on physiology and performance [8,23], yet the processes governing gut microbiome assembly and temporal dynamics remain poorly understood in wild populations [24]. In particular, studies on wild birds—given their unique life-history characteristics [25]–offer a valuable opportunity to examine how age and developmental stages shape gut microbial diversity, composition, and their links to host traits.

In avian species, embryos develop in a sterile egg environment, with gut microbial colonization beginning shortly after hatching. This colonization is influenced by early-life environmental exposure, parental transmission, and diet [26,27]. Initially, the chick gastrointestinal tract is populated by transient bacterial species, which gradually stabilize into an adult-like community [28]. However, investigations into age-related variation in gut microbiota during the chick stage of wild birds remain limited and present inconsistent patterns. For instance, some studies report a decline in microbial diversity with increasing age, as observed in great tits (*Parus major*) [29], while others indicate relatively stable communities throughout development in species such as house sparrows (*Passer domesticus*) and Eurasian kestrels (*Falco tinnunculus*) [30,31]. Additionally, shifts in bacterial abundance have been observed, such as an upward trend in Firmicutes and Bacteroidetes during development of the little penguin (*Eudyptula minor*) [32]. Comparative analyses between chick and adult gut microbiotas are scarce, with no consensus established regarding potential differences [28,30,31,33,34,35]. Notably, previous studies have used different sample types—including feces, cloacal swabs, and intestinal contents—which may partly contribute to the heterogeneous results reported across studies [29,30,31]. Together, these inconsistencies highlight the need for further investigation into the developmental dynamics of avian gut microbiota to clarify age-related community assembly and their implications for host physiology and fitness.

The gut microbiome plays a crucial role in host development and health, with the timing of colonization and community structure recognized as key mechanisms driving developmental plasticity [23,36]. Microbial effects on host traits can occur through contemporary associations, where current microbial diversity or composition relates to host condition, or through time-lagged associations, where early microbial states predict later host traits [29,37,38]. While a more diverse microbiota generally indicates a healthier gut microbiome [39], its relationship with host fitness is complex. For instance, lower gut microbiota diversity is associated with higher body mass index and greater visceral fat [40,41]. Indeed, the composition of the gut microbiota is a more critical determinant of host health than diversity alone, with strain-specific effects, such as those of *Lactobacillus* spp. on fat storage and weight change [42]. A negative time-lagged effect of diversity on subsequent growth was observed in great tit nestlings [38], whereas a positive association between early diversity and scaled mass index was found in yellow-rumped flycatchers (*Ficedula zanthopygia*) [37]. These findings suggest that the gut microbiome–host fitness relationship may vary substantially depending on species, ecological context, or developmental stage. Systematic research is therefore essential to unravel these complex interactions and clarify the functional role of the gut microbiome in wild bird development.

The Japanese tit (*Parus minor*) provides an excellent model for investigating the developmental dynamics of gut microbiota. Its broad geographic distribution and preference for nesting in artificial nest boxes allow for longitudinal sampling under natural conditions. The nestling period (i.e., the age at fledging) typically lasts between 15 and 18 days [43]. Previous research has predominantly focused on mid-to-late nestling stages [29,38], leaving the early stages insufficiently explored, which limits the understanding of how gut microbiota establish and shift throughout development. To address this gap, fecal samples were collected non-invasively from Japanese tit nestlings at four development stages (days 3 to 14) and from breeding adults, while also measuring nestling body condition as a key fitness-related trait. The study focused on the following questions: (i) what are the compositional features of gut microbiota in nestlings and adults; (ii) how do microbial diversity and community structure change across nestling development; (iii) how do nestlings differ from adults in their gut microbiota; and (iv) are microbial diversity or specific taxa abundances linked to nestling body condition? By integrating developmental stages with body condition measures, the study not only characterizes the ontogeny of gut microbiota in a wild passerine, but also provides novel insights into the potential role of microbes in shaping early-life growth.

## 2. Materials and Methods

### 2.1. Study Area and Sample Collection

The field work was conducted in the Zuojia National Nature Reserve (126°0′–126°8′ E, 44°1′–45°0′ N), Jilin, China, from mid-May to mid-June 2022. Artificial nest boxes were hung at a height of approximately 2.5–3 m above the ground to attract wild Japanese tits. These nest boxes were checked every 1–2 days to determine hatching dates, with the day of hatching designated as day 0. Nestlings were individually marked using non-toxic acrylic paint. Nestling fecal samples were collected at four ages: 3, 6, 10, and 14 days post-hatching (hereafter D3, D6, D10, D14), following the same methods in Jin et al. [37]. During each sampling event, nestling body mass and tarsus length were measured to calculate the scaled mass index (SMI), an indicator of body condition. SMI was calculated based on the linear regression of log-body mass on log-tarsus length using type-2 (standardized major axis) regression [44]. In addition, fecal samples were collected from adults during the nestling period following the methods described in Zhang et al. [45], wherein each bird was placed in a cage lined with sterile craft paper to collect naturally excreted feces. Blood samples (20 μL per individual) were collected from the brachial vein and preserved in absolute ethanol at −80 °C. Initially, samples were collected from the same nestlings across six nests at days 3, 6, 10, and 14, as nestlings typically fledge around 15 days after hatching, along with samples from 38 adults across 23 nests. However, nestlings and adults that did not defecate within 10 min were excluded from the study. After quality filtering, the final dataset comprised 95 fecal samples: D3 (n = 12), D6 (n = 19), D10 (n = 21), D14 (n = 18), and adults (n = 25).

### 2.2. Sex Identification

Genomic DNA was extracted from blood samples using the Ezup Column Animal Genomic DNA Purification Kit (Sangon Biotech, Shanghai, China) following the manufacturer’s instructions. The CHD gene regions on both the Z and W chromosomes were amplified using primers P2 (5′-TCTGCATCGCTAAATCCTTT-3′) and P8 (5′-CTCCCAAGGATGAGRAAYTG-3′). PCR products were separated by agarose gel electrophoresis, and fragment sizes were estimated against a DL1000 DNA marker (Takara, Dalian, China). The results were visualized and recorded using the Tanon MINI Space 2000 Gel Imaging System (Tanon, Shanghai, China).

### 2.3. DNA Extractions from Feces

Total microbial genomic DNA was extracted from fecal samples using the Omega Stool DNA Kit (Omega Bio-tek, Norcross, GA, USA) following the manufacturer’s instructions. The concentration and purity of the DNA were determined using a NanoDrop 2000 UV-vis spectrophotometer (Thermo Fisher Scientific, Waltham, MA, USA). DNA integrity was assessed using 1.0% agarose gels. The hypervariable region V3-V4 of the bacterial 16S rRNA gene was amplified with primer pairs 338F (ACTCCTACGGGAGGCAGCAG) and 806R (GGACTACHVGGGTWTCTAAT). The PCR amplification was performed in triplicate for each sample in a 25 μL reaction mixture containing 12.5 μL of 2 × Taq Plus Master Mix, 3 μL of BSA (2 ng/μL), 1 μL each of the forward and reverse primers (5 μM), and 30 ng of template DNA. The volume was adjusted to 25 μL with ddH_2_O. The thermal cycling protocol consisted of an initial denaturation at 95 °C for 5 min; followed by 28 cycles of denaturation at 95 °C for 45 s, annealing at 55 °C for 50 s, and extension at 72 °C for 45 s; with a final extension at 72 °C for 10 min. PCR products were examined by agarose gel electrophoresis and subsequently purified using the Agencourt AMPure XP Nucleic Acid Purification Kit (Beckman Coulter, Brea, CA, USA).

### 2.4. Illumina MiSeq Sequencing

Purified amplicons were quantified using a Qubit 3.0 (Thermo Fisher Scientific, Waltham, MA, USA) fluorometer, and their fragment size distribution was verified by Agilent 2100 Bioanalyzer (Agilent Technologies, Santa Clara, CA, USA). Libraries were then pooled in equimolar concentrations based on this quantification and subjected to paired-end sequencing (2 × 300 bp) on an Illumina MiSeq platform (Model: MiSeq PE300; Illumina, San Diego, CA, USA). All procedures, including library preparation and sequencing, were conducted by Beijing Ovison Gene Technology Co., Ltd. (Beijing, China) following the manufacturer’s standard protocols.

### 2.5. Bioinformatics

Paired-end reads (R1 and R2) were joined using FLASH v1.2.11 [46] with a minimum overlap of 10 bp and a maximum mismatch rate of 0.2. The merged reads were then quality-filtered using Fastp v0.19.6 [47] to remove low-quality bases (Q < 20) and reads containing ambiguous (N) bases. A total of 6,581,554 reads were retained from 95 fecal samples, ranging from 31,156 to 289,663 reads per sample. The processed reads were then imported into QIIME2 v2022.06 [48] for subsequent analyses. Within QIIME2, sequencing errors were removed and Amplicon Sequence Variants (ASVs) were inferred using the Deblur plugin [49], with sequences truncated to 400 bp. Taxonomy was assigned to the ASVs using a Naive Bayes taxonomic classifier trained on the SILVA SSU 138.1 reference database [50]. ASVs classified as mitochondria or chloroplasts, unassigned at the phylum level, present in only one sample, or represented by fewer than 10 reads across the dataset were removed. The final dataset comprised 2,750,107 high-quality reads clustered into 12,006 ASVs, with per-sample read counts ranging from 9036 to 119,528.

To normalize sequencing depth for alpha diversity analyses, the dataset was rarefied to 9036 reads per sample, retaining 11,945 ASVs. To assess whether sequencing depth was sufficient, rarefaction curves (based on observed ASVs; Figure S1) were constructed using the vegan package (v2.7-2). The rarefied ASV table, taxonomy, and phylogenetic tree were imported into R and converted into a phyloseq object using the phyloseq package (v1.50.0) [51]. Observed ASVs and Shannon diversity were obtained using the estimate richness function in phyloseq, and Faith’s phylogenetic diversity (PD) was computed using the pd function in the picante package (v1.8.2). Observed ASVs estimate the richness of distinct ASVs in each sample, Shannon diversity accounts for both richness and evenness, and Faith’s PD reflects the accumulated branch length on the phylogenetic tree, capturing evolutionary diversity. Beta diversity analyses were performed on non-rarefied data and calculated based on relative abundances. Bray–Curtis distances, weighted and unweighted UniFrac distances were computed using the distance function in phyloseq. The phylogenetic tree was produced in QIIME2 using fasttree [52].

### 2.6. Statistical Analysis

All statistical analyses were performed in R v4.4.3 (www.r-project.org). Data visualization was carried out using the ggplot2 v3.5.2 [53] and ggVennDiagram v1.5.4 [54] packages. Relative abundances of bacterial taxa at the phylum and genus levels were calculated and visualized using bar plots.

Age-related changes in gut microbiota, including relative abundances of the top five phyla and alpha diversity metrics (Observed ASVs, Shannon diversity, and Faith’s PD), were analyzed using linear mixed-effects models (LMMs) with the lme4 package (lmer function; v1.1-37). *p* values for fixed effects were derived using the lmerTest package (anova function; v3.1-3) with the Satterthwaite approximation. Model residuals were assessed using the DHARMa package (v0.4.7). In all models, age and sex were included as fixed effects, and Nest ID and Individual ID as random effects. Post hoc pairwise comparisons among age groups were conducted based on the fitted models using Tukey’s adjustment, and *p* values were further corrected for multiple comparisons using the Benjamini–Hochberg false discovery rate (FDR) [55]. Faith’s PD, Bacteroidota and Verrucomicrobiota abundances were log-transformed to improve residual normality.

Linear discriminant analysis (LDA) coupled with effect size measurements (LEfSe), using individual birds as the subject, was used to identify microbial phyla and genera that differed significantly between D14 nestlings and adults [56]. We focused on D14 nestlings because they represent the oldest age group and are the closest in age to adults, making them the most relevant stage for comparison.

Bray–Curtis differences in community composition were visualized using non-metric multidimensional scaling (NMDS), while WU- and UU-based differences were examined using principal coordinate analysis (PCoA). Differences in community structure were tested using permutational multivariate analysis of variance (PERMANOVA; adonis2, 999 permutations), followed by pairwise PERMANOVA between age groups with FDR correction. To evaluate the influence of within-group dispersion, beta diversity dispersion was assessed using PERMDISP (betadisper), with post hoc comparisons performed via Tukey’s Honest Significant Difference (HSD) test.

LMMs were used to examine the relationships between nestling body condition and six microbiome metrics, including three alpha diversity indices (Observed ASVs, Shannon diversity, and Faith’s PD), the relative abundances of the three dominant phyla (Firmicutes, Proteobacteria, and Actinobacteriota), and the core genera (defined as those present in 90% of the samples with a threshold of 0.01% [57]. In cases where significant time-lagged effects were detected at the phylum level, genus-level analyses were subsequently conducted to explore whether specific genera might drive these associations. Two sets of analyses conducted: (a) whether the microbiome at day *t* predicts SMI at the next age stage (hereafter “time-lagged SMI”), and (b) whether the microbiome at day *t* is associated with SMI at the same age (hereafter “contemporary SMI”). For the time-lagged SMI analyses, SMI at the next age stage was used as the response variable, while alpha diversity indices and phylum-level relative abundances at day *t* were included as fixed effects. SMI at day *t* was added as a covariate to control for baseline body condition, and sex was included as an additional fixed effect. Nest ID was incorporated as a random effect. Only individuals with paired measurements at day *t* and the subsequent age stage were included in the time-lagged analyses (D3–6: n = 10; D6–10: n = 16; D10–14: n = 15). For the contemporary SMI analyses, SMI at day *t* was used as the response variable, with the corresponding alpha diversity indices and phylum-level relative abundances at day *t* as fixed effects, together with sex and Nest ID as in the time-lagged models.

The purpose of all analyses was exploratory, aimed at generating rather than confirming hypotheses [58,59]. Therefore, we did not correct *p* values for multiple comparisons, as discussed in Davidson et al. [38].

All data were presented as means ± standard error (SE).

## 3. Results

### 3.1. Gut Microbiota Composition

**Figure 1:**
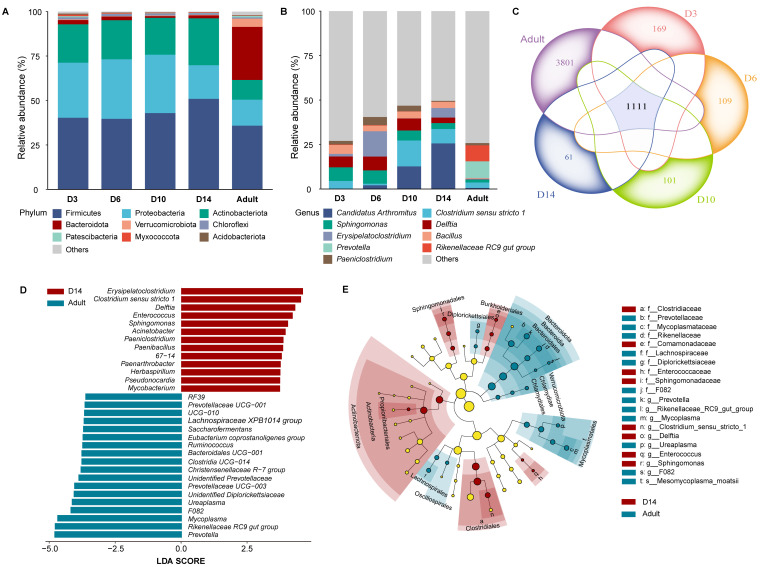
Gut microbiota composition across host ages in Japanese tits. (**A**) Relative abundance of the top nine bacterial phyla across different host ages. (**B**) Relative abundance of the top nine genera across different host ages. (**C**) Venn diagram showing the number of Amplicon Sequence Variants (ASVs) shared and unique across different host ages. Intersection values indicate shared ASVs, whereas values outside intersections indicate unique ASVs. (**D**) Differentially abundant taxa between D14 nestlings and adults identified by LEfSe analysis (Kruskal–Wallis and Wilcoxon tests, *p* < 0.05; LDA score > 3). (**E**) Phylogenetic cladogram of LEfSe identified biomarkers.

A total of 35 bacterial phyla were identified across 95 fecal samples. At the phylum level, the most prominent phyla in nestlings were Firmicutes (43.64% ± 3.74), Proteobacteria (29.10% ± 2.98), Actinobacteriota (22.66% ± 2.37), Bacteroidota (1.66% ± 0.46), and Chloroflexi (1.12% ± 0.16; Figure 1A). In adults, the most prominent phyla were Firmicutes (35.83% ± 4.05), Bacteroidota (29.79% ± 5.09), Proteobacteria (14.61% ± 3.46), Actinobacteriota (11.12% ± 3.23), and Verrucomicrobiota (4.65% ± 2.19; Figure 1A). Results showed that nestling age significantly affected the relative abundances of Actinobacteriota (LMMs, F_4,42_ = 2.57, *p* = 0.047), Bacteroidota (F_4,42_ = 6.03, *p* < 0.001), and Verrucomicrobiota (F_4,42_ = 6.89, *p* < 0.001). Specifically, D6 nestlings had a higher Actinobacteriota abundance compared to adults (adjusted *p* = 0.016), whereas no other age-group comparisons were significant (adjusted *p* ≥ 0.21). For Bacteroidota and Verrucomicrobiota, all nestling age groups exhibited significantly lower abundances than adults (adjusted *p* ≤ 0.028), with no differences were observed among nestling groups. In contrast, Firmicutes showed no age-related variation (F_4,42_ = 0.55, *p* = 0.70). For Proteobacteria, although the overall effect of age was significant (F_4,42_ = 3.11, *p* = 0.019), post hoc tests revealed no significant pairwise differences (adjusted *p* ≥ 0.062). No significant sex-related differences were found for any phyla other than Proteobacteria (Table S1). When the datasets for adults and nestlings were analyzed separately, significant differences were found only between male and female nestlings (F_1,68_ = 4.95, *p* = 0.029; male: 22.07% ± 0.03, female: 35.02% ± 0.05), whereas no significant differences were detected between sexes in adults (F_1,23_ = 0.002, *p* = 0.96).

At the genus level, distinct genera were enriched across different age groups (Figure 1B). In D3 nestlings, *Sphingomonas*, *Delftia*, *Bacillus*, and *Clostridium sensu stricto 1* exhibited higher relative abundances. D6 nestlings were primarily enriched in *Erysipelatoclostridium*, *Delftia*, *Sphingomonas*, and *Paeniclostridium*. For D10 nestlings, enriched genera included *Clostridium sensu stricto 1*, *Candidatus Arthromitus*, *Delftia*, *Sphingomonas*, and *Providencia*. D14 nestlings showed elevated abundances of *Candidatus Arthromitus*, *Clostridium sensu stricto 1*, *Erysipelatoclostridium*, *Bacillus*, *Sphingomonas*, and *Delftia*. In contrast, adults were enriched in *Prevotella*, *Rikenellaceae RC9 gut group*, *Mycoplasma*, and *Clostridium sensu stricto 1*.

Considering the ASVs detected across age groups, both a stable core microbiota and substantial age-specific variation were observed (Figure 1C). A total of 1111 ASVs were shared among all groups, representing a core community maintained throughout host development. In contrast, the number of unique ASVs varied markedly across stages. Adults harbored the highest number of exclusive ASVs (3801), whereas nestlings at D14 exhibited the fewest (61). Nestlings at earlier stages also possessed distinct sets of exclusive ASVs, including 169 at D3, 109 at D6, and 101 at D10. LEfSe analysis results showed 13 genera in D14 nestlings and 18 genera in adults, which were regarded to explain the difference between the two groups (Figure 1D). The D14 nestling was characterized by an abundance of Actinobacteriota, while the adult group was characterized by a higher prevalence of Bacteroidota (Figure 1E).

### 3.2. Alpha Diversity

**Figure 2:**
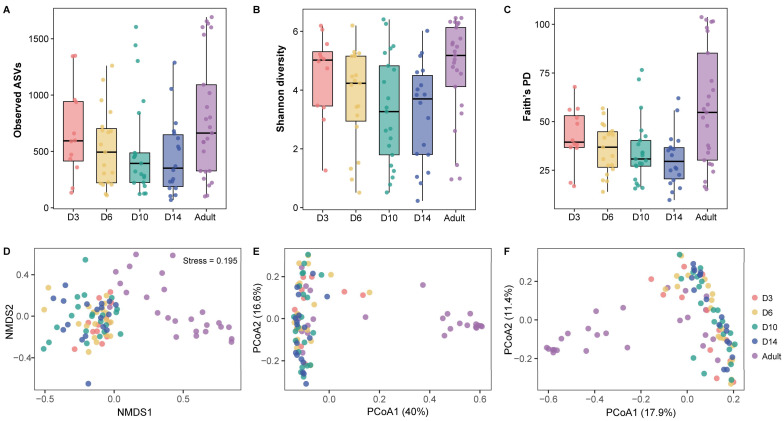
Alpha and beta diversity of gut microbiota across different host ages in Japanese tits. (**A**) Observed ASVs, (**B**) Shannon diversity, and (**C**) Faith’s PD across age groups. (**D**) Non-metric multidimensional scaling (NMDS) ordination based on Bray–Curtis distances. (**E**) Principal coordinates analysis (PCoA) ordination based on weighted UniFrac distances. (**F**) PCoA ordination based on unweighted UniFrac distances. Colors represent different age groups.

Age had a significant effect on Shannon diversity (LMMs, F_4,42_ = 3.21, *p* = 0.016; Figure 2B) and Faith’s PD (F_4,42_ = 4.18, *p* = 0.003; Figure 2C), but not on Observed ASVs (F_4,42_ = 0.88, *p* = 0.49; Figure 2A). Post hoc tests revealed that both Shannon diversity and Faith’s PD were significantly lower in D10 (adjusted *p* ≤ 0.027) and D14 (adjusted *p* ≤ 0.027) compared to adults; Faith’s PD was significantly lower in D6 compared with adults (adjusted *p* = 0.044). In contrast, Shannon diversity and Faith’s PD did not differ significantly among nestling age groups (adjusted *p* ≥ 0.13). In addition, no significant effects of sex were detected (Table S1).

### 3.3. Beta Diversity

Gut microbiota structure varied significantly during the development of Japanese tits. Ordination analyses based on Bray–Curtis (NMDS) as well as weighted and unweighted UniFrac distances (PCoA) revealed a clear separation between adults and nestlings (Figure 2D–F). However, nestlings at different developmental stages largely overlapped, indicating limited differentiation within nestlings (Figure 2D–F). PERMANOVA consistently demonstrated that both age and nest significantly shaped microbial communities across all distance metrics, whereas sex had no detectable effect (Table 1). Pairwise PERMANOVA showed that all nestling age groups differed significantly from adults (adjusted *p* ≤ 0.003). D3 and D6 nestlings were distinct from D14 nestlings under Bray–Curtis (adjusted *p* ≤ 0.012) and weighted UniFrac (adjusted *p* ≤ 0.05), but no other significant differences were observed among nestling groups (adjusted *p* ≥ 0.082). PERMDISP indicated that group dispersion did not confound the differences under Bray–Curtis (*p* = 0.78), although variation in weighted and unweighted UniFrac distances was partly attributable to within-group heterogeneity (*p* ≤ 0.039).

### 3.4. Gut Microbiota and the SMI of Hosts

**Figure 3:**
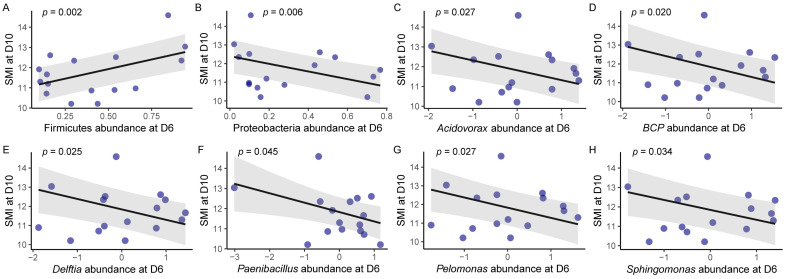
The effect of phylum- (**A**,**B**) and genus-level (**C**–**H**) relative abundance at D6 on time-lagged scaled mass index (SMI) at D10. (**A**) Firmicutes, (**B**) Proteobacteria, (**C**) *Acidovorax*, (**D**) *Burkholderia_Caballeronia_Paraburkholderia* (*BCP*), (**E**) *Delftia*, (**F**) *Paenibacillus*, (**G**) *Pelomonas*, and (**H**) *Sphingomonas*. Blue dots are individual data points, black line is the regression line with 95% confidence intervals (shaded grey). The relative abundance of genus-level is centred and scaled.

In the time-lagged analyses, after controlling for SMI at D6, SMI at D10 showed a positive association with Firmicutes (Figure 3A) and a negative association with Proteobacteria abundance at D6 (Figure 3B), whereas Actinobacteriota abundance did not predict SMI (Firmicutes: β = 1.93, *p* = 0.002; Proteobacteria: β = −2.26, *p* = 0.006; Actinobacteriota: β = −1.13, *p* = 0.38; Table S3). At the genus level, among the 22 core genera present in the nestlings, the relative abundances of *Acidovorax* (β = −0.49, *p* = 0.027), *Burkholderia_Caballeronia_Paraburkholderia* (β = −0.55, *p* = 0.020), *Delftia* (β = −0.54, *p* = 0.025), *Paenibacillus* (β = −0.47, *p* = 0.045), *Pelomonas* (β = −0.55, *p* = 0.027), and *Sphingomonas* (β = −0.50, *p* = 0.034) at D6 negatively predicted SMI at D10 (Figure 3C–H; Table S4). None of the alpha diversity indices were significantly related to time-lagged SMI at any developmental stage (Table S2). Similarly, phylum-level abundances at D3 did not predict SMI at D6, and those at D10 did not predict SMI at D14 (Table S6). Sex had no detectable effect on SMI in any model (Tables S3 and S4). In the contemporary analyses, neither alpha diversity indices nor phylum-level abundances showed significant associations with SMI at any nestling stage. Additionally, sex had no detectable effect on SMI in any model (Tables S5 and S6).

## 4. Discussion

### 4.1. Composition and Dynamics of Gut Microbiota

This study demonstrates that the taxonomic composition of gut microbiota and the relative abundance of microbial phyla differ between nestlings and adults. The observed shifts across developmental stages highlight a dynamic process of microbiome maturation. At phylum level, Firmicutes and Proteobacteria dominated the gut microbiota of nestlings, followed by Actinobacteriota. In contrast, adults were mainly characterized by Firmicutes, Bacteroidota, Proteobacteria, Actinobacteriota, and Verrucomicrobiota. The strong dominance of a few phyla in nestlings indicates a simplified and uneven microbial community, while the more even distribution among multiple phyla in adults suggests increased community complexity and stability as hosts mature. These findings are consistent with previous studies on nestlings [26,31,37] and adults [60,61,62]. The transient elevation of Actinobacteriota in early nestlings (e.g., D6) may reflect initial colonization by environmental bacteria, as many Actinobacteria are Gram-positive taxa that inhabit diverse environments such as soil and air [63,64]. In contrast, the higher relative abundances of Bacteroidota and Verrucomicrobiota in adults likely reflect dietary diversification and the maturation of the gut environment [65,66]. These phyla are typically associated with polysaccharide fermentation and mucin degradation, indicating functional maturation of the gut microbiota [67,68].

At genus level, the gut microbial succession of Japanese tits also followed a distinct developmental trajectory, transitioning from environmentally associated and opportunistic taxa in early nestlings (e.g., *Sphingomonas*, *Delftia*, *Bacillus*) to more host-adapted and functionally specialized genera in adults (e.g., *Prevotella*, *Rikenellaceae RC9 gut group*, *Mycoplasma*). Intermediate stages were characterized by the emergence of anaerobic fermenters such as *Clostridium sensu stricto 1* and *Erysipelatoclostridium*, along with immune-associated taxa like *Candidatus Arthromitus*, indicating the progressive establishment of an anaerobic and immunologically mature gut environment. Similar ontogenetic shifts from environmentally derived to host-adapted microbiota have been widely observed across bird species [28,69,70], suggesting that developmental restructuring of the gut microbiome is a conserved feature of avian microbial ecology.

To identify the specific taxa driving the microbial divergence at the critical developmental juncture of pre-fledging, LEfSe analysis was performed to compare the gut microbiota of D14 nestlings with that of adults. The results revealed 13 and 18 genera as biomarkers for D14 nestlings and adults, respectively (Figure 1D). This divergence was marked by a core phylum-level shift: D14 nestlings were characterized by Actinobacteriota, while adults were dominated by Bacteroidota, underscoring the pivotal phylum-level transition from the end of the nestling period to maturity. The D14 stage represents a critical pre-fledging window [43], indicating the gut microbiota is still transitioning toward the final adult structure and function. This significant restructuring likely plays a vital role in preparing the host for independence, potentially by regulating key physiological processes such as digestion, immunity, and metabolism [27,71].

### 4.2. Age-Related Changes in Gut Microbiota Diversity

Conventionally, gut microbiota diversity is thought to increase progressively during the early stages of life and peak in adulthood [28,72]. In contrast, the results in Japanese tits revealed a different pattern: Shannon and Faith PD indices showed a decreasing trend in nestlings (D3–D14; Figure 2B,C). Adults harbored higher diversity than late-stage nestlings (D10–D14) but were comparable to early-stage nestlings (D3–D6). Newborn nestlings had diverse gut microbiota owing to the rapid colonization of gut microbes shortly after hatching [26,30,31,69]. As nestlings age, microbial diversity tended to decrease. Grond et al. [69] observed a decrease in alpha diversity occurring as early as 3 days after hatching, and Teyssier et al. [29] found a marked reduction in great tits between 8 and 15 days post-hatch. This age-related decline during nestling ontogeny may be explained by several non-exclusive mechanisms, including passive environmental filtering [73], active host selection through immune regulation [74], or community processes such as competitive exclusion [75]. Additionally, physiological changes in morphology [76], metabolism [77], and immunity [78] may further restrict bacterial species. After fledging, however, alpha diversity may increase again as juvenile encounter diverse environments and food sources [79].

Parallel to these alpha diversity shifts, the microbial community structure differed markedly between nestlings and adults. Within the nestling stage, the community structure remains relatively stable overall, but early-stage nestlings (D3–D6) exhibit limited and gradual differentiation from those nearing fledging (D14). This pattern aligns closely with studies on the ontogenetic development of gut microbiota in other avian species [30,31,33]. Consistent with the processes underlying alpha diversity changes, the community structure shifts suggest a transition from random colonization to host specialization. Early nestlings (D3–D6) likely experience stochastic microbial colonization driven by initial environmental exposure [27] and residual yolk sac nutrients [80]. As nestlings grow, the development toward D14 indicates deterministic filtering. Physiological maturation [74] and subtle shifts in parental prey composition [81,82] jointly constrain microbial assembly, eliminating transient species and driving the gradual differentiation. Crucially, the marked structural difference between nestlings and adults represents the completion of this trajectory. The adult gut microbiota is mature, specialized, and stable [35]. This final divergence is driven by the expansion of the dietary niche and the full maturation of host immune and physiological systems, imposing the strongest selection pressure to establish a structurally unique microbial network optimized for adult metabolic demands.

### 4.3. Nest and Sex Effects on Gut Microbiota

In this study, the gut microbial community structure was significantly influenced by the nest effect. Nestlings from the same nest harbored more similar gut microbiota compared to those from different nests. Ordination further revealed that adults and their nestlings clustered more closely together. In altricial birds, such within-nest similarity is common [29,37,38], likely due to the vertical transmission of microbes from parents during feeding [26,83], as well as horizontal transfer through shared food and the nest environment [84,85,86].

In contrast, neither alpha nor beta diversity differed significantly between sexes, suggesting that sexual differentiation has not yet exerted a measurable influence on gut microbial diversity. This absence of a sex effect may reflect the immature endocrine and immune systems of nestlings, as sex-related hormonal modulation of immunity and microbiota is typically established after sexual maturation [27]. Nevertheless, female nestlings exhibited a higher relative abundance of Proteobacteria than males, which may reflect subtle early-life physiological or behavioral differences between sexes that could influence microbial exposure or colonization [87].

### 4.4. Gut Microbiota and Body Condition

Although an increasing number of studies have demonstrated that the microbiome can be an important predictor of host weight or body condition, the microbial profiles and the direction of this relationship vary across species and studies [29,33,37,38,88,89]. For example, a negative relationship between alpha diversity at D8 and body weight at D15 was found in great tits [38], whereas a positive relationship between alpha diversity at D6 and body condition (SMI) at D9 was reported in yellow-rumped flycatchers [37]. Expanding beyond microbial alpha diversity, this study focused on the relative abundance of specific microbial taxa and revealed a novel time-lagged association in Japanese tits: SMI at D10 was positively associated with the relative abundance of Firmicutes and negatively associated with that of Proteobacteria at D6.

Further analyses revealed that the relative abundances of *Acidovorax*, *Burkholderia_Caballeronia_Paraburkholderia*, *Delftia*, *Paenibacillus*, *Pelomonas*, and *Sphingomonas* at D6 negatively predicted SMI at D10. Most of these genera (except *Paenibacillus*, which belongs to Firmicutes) are affiliated with Proteobacteria. The positive effect of Firmicutes may reflect their role in efficient energy harvest through short-chain fatty acid (SCFA) production, which supports host growth and metabolism [90], while Proteobacteria are often associated with gut inflammation and dysbiosis, potentially impairing nutrient absorption and body condition in animals and birds [91]. These findings indicate that the gut microbiota may act as a regulatory mechanism influencing adaptive phenotypic changes in response to environmental cues. Such time-lagged associations may reflect the existence of “critical developmental windows”, during which the microbiota can exert lasting effects on host phenotype–a phenomenon well documented in clinical microbiome research [23,92,93]. In contrast to microbiota composition, no discernible connection was detected between SMI and gut microbiota diversity, whether contemporary or time-lagged, as microbiota diversity is influenced by multiple variables. These complex interactions may obscure direct links between host body condition and microbiota diversity [27,71,94].

Although this study offers useful findings, several limitations should be acknowledged. First, although the sample sizes at each developmental stage were adequate for revealing general patterns, they were constrained by the practical challenges of working with a wild population and the ethical requirement to minimize animal handling. Second, to avoid excessive disturbance to fledglings and prevent potential impacts on the breeding population, we restricted sampling to the nestling period. As a result, our findings primarily characterize the early establishment of the gut microbiota prior to fledging and independence.

## 5. Conclusions

This study provides an integrative view of gut microbiota development in wild Japanese tit nestlings, capturing both temporal shifts and fitness-related correlates across early growth stages. Using a longitudinal sampling design, the results demonstrate that age is a dominant factor structuring gut microbial diversity and composition, with nestlings displaying pronounced, stage-specific microbial transitions before acquiring a distinct adult-like community enriched in Bacteroidota and Verrucomicrobiota. The influence of the nesting environment was also evident, as nest identity contributed to variation in community structure, highlighting the shared microbial context experienced by siblings. Nestling body condition emerged as a meaningful correlate of microbiota composition: early-life abundances of Firmicutes and Proteobacteria predicted subsequent body condition, suggesting that microbial trajectories during key developmental windows may shape physiological performance. Overall, these findings emphasize the ecological relevance of early host–microbe interactions and their potential role in avian growth and fitness.

## Figures and Tables

**Table 1:** The effects of age, sex, and nest on the composition of gut microbial communities in Japanese tits based on permutational multivariate analysis of variances (PERMANOVA) with Bray–Curtis, weighted and unweighted UniFrac distances. Significance: * *p* < 0.05.

Distance Matrix	Variable	*df*	R^2^	*F* Value	*p* Value
Bray–Curtis	age	4	0.123	3.59	0.001 *
	sex	1	0.010	1.20	0.188
	nest	21	0.285	1.59	0.001 *
Weighted UniFrac	age	4	0.241	9.86	0.001 *
	sex	1	0.007	1.12	0.315
	nest	21	0.335	2.61	0.001 *
Unweighted UniFrac	age	4	0.139	4.18	0.001 *
	sex	1	0.007	0.86	0.621
	nest	21	0.287	1.64	0.001 *

## Data Availability

Data available in a publicly accessible repository. The data presented in this study are openly available in the NCBI database at [https://www.ncbi.nlm.nih.gov/], reference number [PRJNA1356682].

## Supplementary Materials

The following supporting information can be downloaded at: https://www.mdpi.com/article/10.3390/microorganisms13122840/s1, Figure S1. Rarefaction curves of observed ASVs across all samples. Table S1. Results of linear mixed-effects models (LMMs) testing the effects of age and sex on gut microbiota phylum-level relative abundance and alpha diversity indices. * *p* < 0.05. Table S2. LMM results from time-lagged scaled mass index (SMI) analyses testing the effects of the alpha diversity (Observed ASVs, Shannon, and Faith’s PD). (a–c) Effects of D3 alpha diversity on SMI at D6, (d–f) Effects of D6 alpha diversity on SMI at D10 and (g–i) Effects of D10 alpha diversity on SMI at D14. * *p* < 0.05. Table S3. LMM results from time-lagged scaled mass index (SMI) analyses testing the effects of the relative abundance of the three most abundant phyla (Proteobacteria, Firmicutes, and Actinobacteriota). (a–c) Effects of D3 abundance on SMI at D6, (d–f) Effects of D6 abundance on SMI at D10. (g–i) Effects of D10 abundance on SMI at D14. * *p* < 0.05. Table S4. LMM results from time-lagged SMI (D10) analyses testing the effects of the relative abundance of the most abundant genera at D6. * *p* < 0.05. Table S5. LMM results for the effect of alpha diversity (Observed ASVs, Shannon, and Faith’s PD) on SMI. (a–c) D3 contemporary SMI, (d–f) D6 contemporary SMI, (g–i) D10 contemporary SMI and (j–l) D14 contemporary SMI. * *p* < 0.05. Table S6. LMM results for the effect of relative abundance of the three most abundant phyla (Firmicutes, Proteobacteria, and Actinobacteriota) on SMI. (a–c) D3 contemporary SMI, (d–f) D6 contemporary SMI, (g–i) D10 contemporary SMI and (j–l) D14 contemporary SMI. * *p* < 0.05.
