# Supplementary material for: Dynamics of Gut Microbiota in Japanese Tits (Parus minor) Across Developmental Stages: Composition, Diversity, and Associations with Body Condition
Source: Microorganisms. 2025 Dec 14;13(12):2840. doi: 10.3390/microorganisms13122840 (PMC12736322; doi:10.3390/microorganisms13122840)
Supplement: Supplementary file 1 [file microorganisms-13-02840-s001.zip › microorganisms-4020293-supplementary.pdf]

microorganisms

MDPI

Supplementary Materials

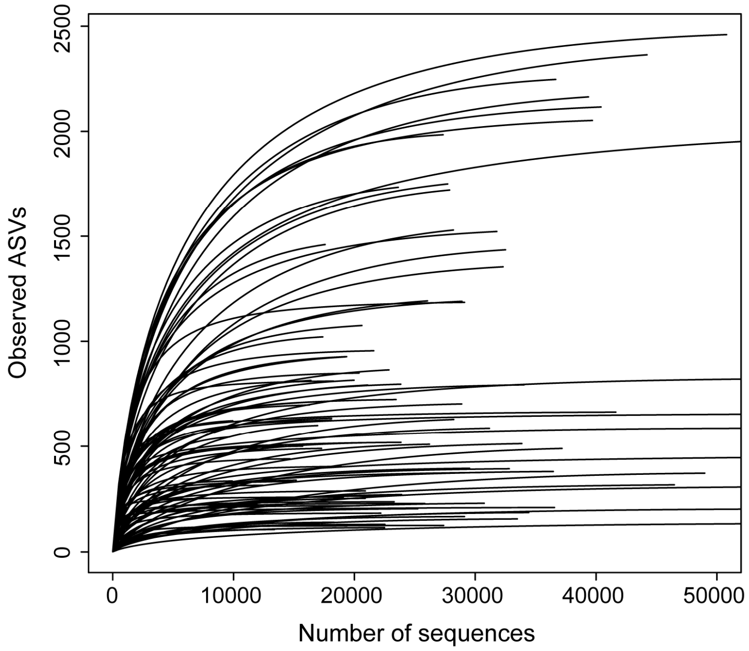

Figure S1. Rarefaction curves of observed ASVs across all samples.

Table S1. Results of linear mixed-effects models (LMMs) testing the effects of age and sex on gut microbiota phylum-level relative abundance and alpha diversity indices. * $p < 0.05$.

Dependent variable	Independent variable	*F* value	*p* value
Firmicutes	age	0.552	0.698
	sex	0.001	0.980
Proteobacteria	age	3.112	0.019*
	sex	4.382	0.039*
Actinobacteriota	age	2.569	0.047*
	sex	1.824	0.180
Bacteroidota	age	6.030	<0.001*
	sex	0.121	0.729
Verrucomicrobiota	age	6.889	<0.001*
	sex	2.722	0.103
Obversed ASVs	age	0.879	0.486
	sex	0.014	0.905
Shannon diversity	age	3.209	0.016*
	sex	0.143	0.706
Faith's PD	age	4.178	0.006*
	sex	0.013	0.910

 https://doi.org/10.3390/microorganisms13122840

Table S2. LMM results from time-lagged scaled mass index (SMI) analyses testing the effects of the alpha diversity (Observed ASVs, Shannon, and Faith's PD). (a–c) Effects of D3 alpha diversity on SMI at D6, (d–f) Effects of D6 alpha diversity on SMI at D10 and (g–i) Effects of D10 alpha diversity on SMI at D14. * $p < 0.05$

**Dependent/Independent variable**	**Estimate**	**SE**	***df***	***t* value**	***p* value**
(a) Nestling SMI (time-lagged)					
(Intercept)	7.910	0.912	8.674	6	<0.001*
Observed ASVs (D3)	−0.170	0.125	−1.365	6	0.221
SMI (D3)	−0.269	0.310	−0.868	6	0.419
sex	−0.120	0.305	−0.393	6	0.708
(b) Nestling SMI (time-lagged)					
(Intercept)	7.709	0.969	7.959	6	<0.001*
Shannon (D3)	−0.209	0.245	−0.852	6	0.427
SMI (D3)	−0.176	0.321	−0.550	6	0.602
sex	0.003	0.314	0.009	6	0.993
(c) Nestling SMI (time-lagged)					
(Intercept)	8.165	0.920	8.877	6	<0.001*
Faith's PD (D3)	−0.217	0.133	−1.634	6	0.153
SMI (D3)	−0.345	0.308	−1.120	6	0.306
sex	−0.041	0.278	−0.147	6	0.888
(d) Nestling SMI (time-lagged)					
(Intercept)	6.734	4.076	1.652	9.618	0.131
Observed ASVs (D6)	−0.132	0.304	−0.436	8.531	0.674
SMI (D6)	0.695	0.563	1.234	9.434	0.247
sex	0.027	0.521	0.051	9.914	0.960
(e) Nestling SMI (time-lagged)					
(Intercept)	8.305	3.378	2.459	9.206	0.036
Shannon (D6)	−0.359	0.211	−1.702	7.241	0.131
SMI (D6)	0.483	0.466	1.037	8.925	0.327
sex	0.036	0.454	0.080	8.975	0.938
(f) Nestling SMI (time-lagged)					
(Intercept)	6.604	4.080	1.619	10.524	0.135
Faith's PD (D6)	−0.081	0.309	−0.263	8.532	0.799
SMI (D6)	0.715	0.561	1.273	10.276	0.231
sex	0.010	0.520	0.019	9.842	0.985
(g) Nestling SMI (time-lagged)					
(Intercept)	13.959	1.759	7.938	10.988	<0.001*
Observed ASVs (D10)	0.089	0.189	0.469	7.579	0.652
SMI (D10)	0.007	0.149	0.048	10.967	0.962
sex	0.029	0.423	0.068	9.418	0.948
(h) Nestling SMI (time-lagged)					
(Intercept)	13.975	1.644	8.502	10.529	<0.001*
Shannon (D10)	0.227	0.170	1.333	7.574	0.221
SMI (D10)	0.017	0.139	0.121	10.522	0.906
sex	−0.093	0.389	−0.239	8.372	0.817
(i) Nestling SMI (time-lagged)					
(Intercept)	14.326	1.788	8.012	10.868	<0.001*
Faith's PD (D10)	0.200	0.232	0.861	7.358	0.416
SMI (D10)	−0.015	0.148	−0.099	10.913	0.923
sex	−0.060	0.426	−0.142	9.206	0.890

Table S3. LMM results from time-lagged scaled mass index (SMI) analyses testing the effects of the relative abundance of the three most abundant phyla (Proteobacteria, Firmicutes, and Actinobacteriota). (a–c) Effects of D3 abundance on SMI at D6, (d–f) Effects of D6 abundance on SMI at D10. (g–i) Effects of D10 abundance on SMI at D14. * $p < 0.05$

Dependent/Independent variable	Estimate	SE	*df*	*t* value	*p* value
(a) Nestling SMI (time-lagged)					
(Intercept)	7.309	0.914	7.993	6	<0.001*
Firmicutes (D3)	0.980	1.192	0.821	6	0.443
SMI (D3)	−0.192	0.326	−0.589	6	0.578
sex	−0.131	0.359	−0.366	6	0.727
(b) Nestling SMI (time-lagged)					
(Intercept)	7.392	0.941	7.856	6	<0.001*
Proteobacteria (D3)	0.411	0.916	0.449	6	0.669
SMI (D3)	−0.174	0.344	−0.507	6	0.630
sex	−0.006	0.329	−0.020	6	0.985
(c) Nestling SMI (time-lagged)					
(Intercept)	8.056	0.991	8.132	6	<0.001*
Actinobacteriota (D3)	−0.914	0.722	−1.266	6	0.252
SMI (D3)	−0.267	0.317	−0.844	6	0.431
sex	−0.161	0.325	−0.497	6	0.637
(d) Nestling SMI (time-lagged)					
(Intercept)	6.023	1.942	3.101	8.215	0.014*
Firmicutes (D6)	1.929	0.390	4.948	6.952	0.002*
SMI (D6)	0.671	0.261	2.576	7.534	0.035*
sex	0.162	0.268	0.604	7.559	0.564
(e) Nestling SMI (time-lagged)					
(Intercept)	5.114	2.324	2.200	8.020	0.059
Proteobacteria (D6)	−2.258	0.581	−3.890	6.913	0.006*
SMI (Day-6)	0.988	0.323	3.059	7.464	0.017*
sex	0.378	0.328	1.155	7.610	0.283
(f) Nestling SMI (time-lagged)					
(Intercept)	7.005	3.691	1.898	9.285	0.089
Actinobacteriota (D6)	−1.126	1.218	−0.925	7.898	0.383
SMI (D6)	0.696	0.501	1.388	9.348	0.197
sex	−0.033	0.506	−0.064	9.870	0.950
(g) Nestling SMI (time-lagged)					
(Intercept)	13.844	1.774	7.802	10.742	<0.001*
Firmicutes (D10)	−0.052	0.518	−0.100	10.193	0.922
SMI (D10)	0.012	0.150	0.082	10.890	0.936
sex	0.123	0.383	0.321	9.437	0.755
(h) Nestling SMI (time-lagged)					
(Intercept)	14.017	1.808	7.753	10.798	<0.001*
Proteobacteria (D10)	−0.191	0.632	−0.303	10.967	0.768

SMI (D10)	0.004	0.150	0.023	10.990	0.982
sex	0.080	0.401	0.201	9.918	0.845
(i) Nestling SMI (time-lagged)					
(Intercept)	13.891	1.758	7.902	10.955	<0.001*
Actinobacteriota (D10)	0.353	0.864	0.408	7.370	0.695
SMI (D10)	0.004	0.149	0.025	10.923	0.980
sex	0.065	0.403	0.161	9.538	0.876

Table S4 LMM results from time-lagged SMI (D10) analyses testing the effects of the relative abundance of the most abundant genera at D6. * $p < 0.05$

**Dependent/Independent variable**	**Estimate**	**SE**	***df***	***t* value**	***p* value**
(a) Nestling SMI (time-lagged)					
(Intercept)	5.780	3.649	1.584	10.588	0.143
67-14	−0.177	0.253	−0.702	9.387	0.500
SMI (D6)	0.823	0.504	1.632	10.357	0.133
Sex	0.088	0.520	0.170	10.268	0.868
(b) Nestling SMI (time-lagged)					
(Intercept)	5.709	2.725	2.095	8.208	0.069
Acidovorax	−0.493	0.178	−2.764	7.179	0.027*
SMI (D6)	0.846	0.374	2.261	7.794	0.054
Sex	−0.006	0.371	−0.017	7.946	0.987
(c) Nestling SMI (time-lagged)					
(Intercept)	5.099	3.172	1.607	7.504	0.149
Acinetobacter (D6)	−0.458	0.230	−1.991	7.392	0.085
SMI (D6)	0.935	0.438	2.135	7.194	0.069
Sex	−0.092	0.418	−0.219	7.948	0.832
(d) Nestling SMI (time-lagged)					
(Intercept)	6.310	3.684	1.713	9.992	0.118
Bacillus (D6)	−0.109	0.253	−0.431	8.864	0.677
SMI (D6)	0.754	0.509	1.480	9.743	0.171
Sex	0.046	0.520	0.088	9.575	0.932
(e) Nestling SMI (time-lagged)					
(Intercept)	6.227	3.116	1.999	8.910	0.077
Bradyrhizobium (D6)	−0.391	0.204	−1.916	6.937	0.097
SMI (D6)	0.753	0.429	1.754	8.646	0.115
Sex	0.247	0.456	0.541	8.651	0.602
(f) Nestling SMI (time-lagged)					
(Intercept)	5.983	2.598	2.303	7.820	0.051
Burkholderia Caballeronia Paraburkholderia	−0.554	0.186	−2.984	7.224	0.020*
SMI (D6)	0.805	0.355	2.272	7.242	0.056
Sex	0.100	0.355	0.283	7.342	0.785
(g) Nestling SMI (time-lagged)					
(Intercept)	6.497	2.644	2.457	8.056	0.039*
Delftia	−0.539	0.193	−2.796	7.339	0.025*
SMI (D6)	0.735	0.362	2.032	7.532	0.079
Sex	0.061	0.366	0.167	7.605	0.871
(h) Nestling SMI (time-lagged)					
(Intercept)	5.797	3.400	1.705	8.950	0.123
Erysipelatoclostridium (D6)	0.336	0.247	1.361	8.004	0.211
SMI (D6)	0.817	0.469	1.742	8.734	0.116
Sex	0.111	0.481	0.232	9.086	0.822

(i) Nestling SMI (time-lagged)					
(Intercept)	5.190	3.604	1.440	7.104	0.192
Escherichia-Shigella (D6)	−0.316	0.254	−1.245	7.131	0.253
SMI (D6)	0.906	0.495	1.831	6.801	0.111
Sex	0.040	0.462	0.086	7.784	0.934
(j) Nestling SMI (time-lagged)					
(Intercept)	6.379	2.921	2.184	8.208	0.060
Herbaspirillum (D6)	−0.449	0.205	−2.192	7.411	0.062
SMI (D6)	0.752	0.401	1.873	7.827	0.099
Sex	0.016	0.405	0.038	7.977	0.970
(k) Nestling SMI (time-lagged)					
(Intercept)	6.279	3.703	1.695	9.845	0.121
IMCC26256 (D6)	−0.090	0.245	−0.365	7.808	0.725
SMI (D6)	0.757	0.513	1.476	9.628	0.172
Sex	0.044	0.522	0.085	9.821	0.934
(l) Nestling SMI (time-lagged)					
(Intercept)	5.896	3.391	1.739	9.200	0.115
Methylobacterium-Methylorubrum (D6)	−0.313	0.242	−1.293	8.077	0.232
SMI (D6)	0.811	0.468	1.735	9.013	0.117
Sex	0.120	0.478	0.250	8.445	0.808
(m) Nestling SMI (time-lagged)					
(Intercept)	6.835	3.542	1.930	9.458	0.084
Microbacterium (D6)	−0.235	0.244	−0.965	8.077	0.363
SMI (D6)	0.683	0.489	1.395	9.246	0.196
Sex	−0.017	0.495	−0.035	9.613	0.973
(n) Nestling SMI (time-lagged)					
(Intercept)	6.198	3.656	1.695	9.991	0.121
Mycobacterium (D6)	−0.102	0.265	−0.385	8.985	0.709
SMI (D6)	0.766	0.506	1.515	9.740	0.162
Sex	0.072	0.535	0.135	9.906	0.896
(o) Nestling SMI (time-lagged)					
(Intercept)	6.060	3.697	1.639	9.073	0.135
Nakamurella (D6)	0.083	0.273	0.303	8.327	0.769
SMI (D6)	0.787	0.510	1.542	8.892	0.158
Sex	0.037	0.521	0.072	9.358	0.944
(p) Nestling SMI (time-lagged)					
(Intercept)	4.970	3.867	1.285	9.364	0.230
Nocardioides (D6)	0.216	0.277	0.782	7.573	0.458
SMI (D6)	0.928	0.531	1.749	9.135	0.114
Sex	0.153	0.536	0.284	9.904	0.782
(q) Nestling SMI (time-lagged)					
(Intercept)	5.865	2.973	1.973	9.408	0.079
Paenibacillus	−0.471	0.200	−2.358	8.238	0.045*
SMI (D6)	0.821	0.410	2.001	9.190	0.076

Sex	0.046	0.416	0.110	9.135	0.915
(r) Nestling SMI (time-lagged)					
(Intercept)	5.423	2.752	1.971	8.201	0.083
Pelomonas	−0.548	0.199	−2.756	7.393	0.027*
SMI (D6)	0.878	0.377	2.330	7.778	0.049
Sex	0.110	0.372	0.297	7.777	0.774
(s) Nestling SMI (time-lagged)					
(Intercept)	6.617	3.377	1.959	8.610	0.083
Pseudomonas (D6)	−0.252	0.232	−1.085	7.283	0.313
SMI (D6)	0.710	0.465	1.526	8.330	0.164
Sex	0.064	0.479	0.134	8.279	0.896
(t) Nestling SMI (time-lagged)					
(Intercept)	5.866	3.694	1.588	9.254	0.146
Romboutsia (D6)	−0.194	0.331	−0.585	11.992	0.569
SMI (D6)	0.824	0.514	1.603	9.157	0.143
Sex	−0.079	0.525	−0.150	11.301	0.883
(u) Nestling SMI (time-lagged)					
(Intercept)	6.763	2.705	2.500	7.921	0.037*
Sphingomonas	−0.503	0.193	−2.606	7.343	0.034*
SMI (D6)	0.701	0.370	1.894	7.385	0.098
Sex	0.033	0.376	0.088	7.491	0.932
(v) Nestling SMI (time-lagged)					
(Intercept)	7.564	3.417	2.214	8.912	0.054
Streptomyces (D6)	−0.317	0.252	−1.262	8.638	0.240
SMI (D6)	0.576	0.473	1.218	8.671	0.255
Sex	0.071	0.475	0.150	9.300	0.884

Table S5. LMM results for the effect of alpha diversity (Observed ASVs, Shannon, and Faith's PD) on SMI. (a–c) D3 contemporary SMI, (d–f) D6 contemporary SMI, (g–i) D10 contemporary SMI and (j–l) D14 contemporary SMI. * $p < 0.05$

Dependent/Independent variable	Estimate	SE	*df*	*t* value	*p* value
(a) Nestling SMI (D3)					
(Intercept)	2.851	0.144	9.000	19.794	<0.001*
Observed ASVs	−0.093	0.117	9.000	−0.793	0.448
sex	−0.265	0.273	9.000	−0.972	0.357
(b) Nestling SMI (D3)					
(Intercept)	2.771	0.179	5.056	15.515	<0.001*
Shannon	0.068	0.150	8.972	0.457	0.659
sex	−0.184	0.273	8.918	−0.675	0.517
(c) Nestling SMI (D3)					
(Intercept)	2.854	0.149	4.664	19.187	<0.001*
Faith's PD	−0.106	0.120	7.620	−0.885	0.403
sex	−0.216	0.265	8.940	−0.812	0.438
(d) Nestling SMI (D6)					
(Intercept)	7.1878	0.200	7.451	35.896	<0.001*
Observed ASVs	−0.181	0.133	13.024	−1.359	0.197
sex	0.071	0.242	14.103	0.293	0.774
(e) Nestling SMI (D6)					
(Intercept)	7.181	0.210	7.379	34.264	<0.001*
Shannon	−0.090	0.122	11.873	−0.739	0.474
sex	0.114	0.247	14.430	0.461	0.652
(f) Nestling SMI (D6)					
(Intercept)	7.208	0.213	7.391	33.778	<0.001*
Faith's PD	−0.184	0.134	12.080	−1.373	0.195
sex	0.060	0.239	13.917	0.250	0.806
(g) Nestling SMI (D10)					
(Intercept)	11.625	0.615	7.043	18.908	<0.001*
Observed ASVs	−0.022	0.218	14.901	−0.102	0.920
sex	0.248	0.544	15.944	0.455	0.655
(h) Nestling SMI (D10)					
(Intercept)	11.656	0.614	7.324	18.988	<0.001*
Shannon	0.052	0.230	14.335	0.227	0.823
sex	0.214	0.535	15.323	0.400	0.695
(i) Nestling SMI (D10)					
(Intercept)	11.649	0.618	7.090	18.846	<0.001*
Faith's PD	0.037	0.222	14.887	0.166	0.870
sex	0.210	0.542	15.965	0.387	0.704
(j) Nestling SMI (D14)					
(Intercept)	13.576	0.408	8.199	33.262	<0.001*
Observed ASVs	0.128	0.236	12.896	0.542	0.597

sex	0.491	0.411	12.879	1.193	0.254
(k) Nestling SMI (D14)					
(Intercept)	13.559	0.410	7.339	33.032	<0.001*
Shannon	0.058	0.258	14.833	0.224	0.826
sex	0.494	0.434	14.220	1.136	0.275
(l) Nestling SMI (D14)					
(Intercept)	13.585	0.411	8.520	33.086	<0.001*
Faith’s PD	0.081	0.227	13.166	0.359	0.725
sex	0.469	0.412	12.953	1.138	0.276

Table S6. LMM results for the effect of relative abundance of the three most abundant phyla (Firmicutes, Proteobacteria, and Actinobacteriota) on SMI. (a–c) D3 contemporary SMI, (d–f) D6 contemporary SMI, (g–i) D10 contemporary SMI and (j–l) D14 contemporary SMI. * $p < 0.05$

Dependent/Independent variable	Estimate	SE	*df*	*t* value	*p* value
(a) Nestling SMI (D3)					
(Intercept)	2.873	0.264	4.823	10.890	<0.001*
Firmicutes	−0.183	0.531	8.991	−0.345	0.738
sex	−0.164	0.282	8.193	−0.581	0.577
(b) Nestling SMI (D3)					
(Intercept)	2.607	0.249	8.980	10.462	<0.001*
Proteobacteria	0.646	0.665	8.672	0.972	0.357
sex	−0.204	0.263	8.995	−0.777	0.457
(c) Nestling SMI (D3)					
(Intercept)	2.947	0.215	9.000	13.711	<0.001*
Actinobacteriota	−0.544	0.667	9.000	−0.815	0.436
sex	−0.306	0.285	9.000	−1.076	0.310
(d) Nestling SMI (D6)					
(Intercept)	7.264	0.279	13.870	26.035	<0.001*
Firmicutes	−0.219	0.410	12.044	−0.535	0.602
sex	0.101	0.253	14.345	0.399	0.696
(e) Nestling SMI (D6)					
(Intercept)	7.022	0.233	10.362	30.077	0.000
Proteobacteria	0.578	0.429	11.761	1.346	0.204
sex	0.004	0.253	13.896	0.015	0.988
(f) Nestling SMI (D6)					
(Intercept)	7.238	0.261	13.496	27.753	0.000
Actinobacteriota	−0.296	0.652	12.846	−0.455	0.657
sex	0.104	0.255	14.272	0.406	0.690
(g) Nestling SMI (D10)					
(Intercept)	11.700	0.699	9.018	16.729	0.000
Firmicutes	−0.162	0.855	17.226	−0.190	0.852
sex	0.246	0.530	15.081	0.464	0.649
(h) Nestling SMI (D10)					
(Intercept)	11.694	0.711	10.013	16.449	0.000
Proteobacteria	−0.146	0.889	15.246	−0.164	0.872
sex	0.208	0.538	14.881	0.387	0.704
(i) Nestling SMI (D10)					
(Intercept)	11.550	0.637	8.679	18.143	0.000
Actinobacteriota	0.511	1.261	13.742	0.405	0.692
sex	0.201	0.532	15.392	0.378	0.711
(j) Nestling SMI (D14)					
(Intercept)	13.590	0.518	11.122	26.226	0.000
Firmicutes	−0.074	0.799	13.785	−0.093	0.927

sex	0.473	0.435	14.336	1.089	0.294
(k) Nestling SMI (D14)					
(Intercept)	13.595	0.525	11.100	25.909	0.000
Proteobacteria	−0.148	1.526	14.990	−0.097	0.924
sex	0.452	0.418	13.395	1.081	0.299
(l) Nestling SMI (D14)					
(Intercept)	13.491	0.520	10.974	25.947	0.000
Actinobacteriota	0.220	1.022	14.828	0.215	0.833
sex	0.485	0.430	14.153	1.130	0.277
